# Efficacy, effectiveness and immunogenicity of reduced HPV vaccination schedules: A review of available evidence

**DOI:** 10.14745/ccdr.v50i06a01

**Published:** 2024-06-28

**Authors:** Joshua Montroy, Marina I Salvadori, Nicole Forbes, Vinita Dubey, Sarah Almasri, Anna Jirovec, Cathy Yan, Katarina Gusic, Adrienne Stevens, Kelsey Young, Matthew Tunis

**Affiliations:** 1 Centre for Immunization Programs, Public Health Agency of Canada, Ottawa, ON; 2 Toronto Public Health and University of Toronto Dalla Lana School of Public Health, Toronto, ON

**Keywords:** HPV, vaccination, dose-reduction, dosing schedule, effectiveness, cancer, evidence review

## Abstract

**Background:**

Current National Advisory Committee on Immunization (NACI) guidance recommends human papillomavirus (HPV) vaccines be administered as a two or three-dose schedule. Recently, several large clinical trials have reported the clinical benefit of a single HPV vaccine dose. As a result, the World Health Organization released updated guidance on HPV vaccines in 2022, recommending a two-dose schedule for individuals aged 9–20 years, and acknowledging the use of an alternative off-label single dose schedule.

**Objective:**

The objective of this overview is to provide a detailed account of the available evidence comparing HPV vaccination schedules, which was considered by NACI when updating recommendations on HPV vaccines.

**Methods:**

To identify relevant evidence, existing systematic reviews were leveraged where possible. Individual studies were critically appraised, and the Grading of Recommendations Assessment, Development and Evaluation (GRADE) methodology was used to assess the certainty of evidence.

**Results:**

Available evidence suggests that a one, two, or three-dose HPV vaccine schedule may provide similar protection from HPV infection. While antibody levels against HPV vaccine types were statistically significantly lower with a single dose schedule compared to two or three doses, titres were sustained for up to 16 years. The clinical significance of lower antibody titres is unknown, as there is no established immunologic correlate of protection.

**Conclusion:**

While the available evidence on single-dose HPV vaccination schedules shows a one-dose schedule is highly effective, continued follow-up of single-dose cohorts will be critical to understanding the relative duration of protection for reduced dose schedules and informing future NACI guidance on HPV vaccines.

## Introduction

Human papillomavirus (HPV) infections are the causative agent of several cancers, including virtually all cervical cancers, other anogenital cancers, as well as head and neck cancers and anogenital warts (AGW) ((1,2)). HPV vaccines were first authorized in 2006 and have been shown to be highly effective (3,4). In Canada, a two or three-dose schedule is recommended for healthy individuals aged 9–14 years, and a three-dose schedule is recommended for healthy individuals aged 15 years and over, and for immunocompromised individuals (5). Recently, the World Health Organization (WHO) released an updated position paper on HPV vaccination schedules, detailing that while a two-dose schedule is recommended for those over 9 years of age, an alternative off-label, single-dose schedule can be used in those aged 9–20 years ((6)). Several other jurisdictions, such as the United Kingdom, have since updated their HPV vaccination recommendations to include a single-dose schedule ((7–9)). This updated guidance was based on several factors, including emerging evidence indicating that a single dose of HPV vaccine provides similar levels of protection from HPV infection as multi-dose schedules ((10)).

Canadian provinces and territories have asked that the National Advisory Committee on Immunization (NACI) review the currently available evidence and potentially provide updated guidance on reduced HPV immunization schedules. The Public Health Agency of Canada (PHAC) has prepared this overview to review the available clinical evidence on reduced HPV vaccination schedules (with a focus on single-dose schedules), with an objective to help inform NACI evidence-informed recommendations and decision-making for vaccine programs in Canada.

## Methods

**Table 1** outlines eligibility criteria for studies included in this analysis. To identify relevant studies, an update of a 2022 systematic review ((10)) performed by Cochrane Response in collaboration with the Strategic Advisory Group of Experts on Immunization (SAGE) (which itself was a modified update of a previous Cochrane Response review ((11))) was performed. The updated literature search allowed for identification of any additional studies published since 2022 or any available updated data from included studies (e.g., both recent publications and proceedings from international conferences).

**Table 1:** Study eligibility criteria

Criteria	Eligibility (one vs. two/three doses)	Eligibility (two vs. three doses)
Population	Individuals ≥9 years of age	
Intervention	One dose of GARDASIL®9 or CERVARIX®. Considering limitations to evidence (e.g., limited follow-up time) on GARDASIL®9, indirect evidence from studies using GARDASIL®4 was also considered.	Two doses of GARDASIL®9 or CERVARIX®. Considering limitations to evidence (e.g., limited follow-up time) on GARDASIL®9, indirect evidence from studies using GARDASIL®4 was also considered.
Comparator	Two or three doses of GARDASIL®9 or CERVARIX® (with the interval between the first and last dose in the series being at least six months). Considering limitations to evidence (e.g., limited follow-up time) on GARDASIL®9, indirect evidence from studies using GARDASIL®4 was also considered. Note: While not directly comparing the clinical benefit of HPV vaccines by the number of doses, studies evaluating the immunogenicity or vaccine efficacy/effectiveness of a one-dose HPV vaccine schedule compared to no HPV vaccine were also included.	Three doses of GARDASIL®9 or CERVARIX®. Considering limitations to evidence (e.g., limited follow-up time) on GARDASIL®9, indirect evidence from studies using GARDASIL®4 was also considered.
Outcomes	Outcomes rated as critical for decision-making (deemed equally critical): · HPV-associated cancers · CIN2+ · Histological and/or cytological abnormalities (including CIN1) · Infection with vaccine-associated serotypes o HPV vaccine type antibody titres Outcomes rated as important for decision-making (deemed equally important): · Anogenital warts o Juvenile onset recurrent respiratory papillomatosis (JORPP)	
Study design	Randomized controlled trials, non-randomized trials, and observational studies. Observational studies assessed to be at a serious or critical risk of bias were excluded.	

Abbreviations: CIN, cervical intraepithelial neoplasia; HPV, human papilloma virus

For analyses comparing a single dose to zero, two, or three doses, the Grading of Recommendations Assessment, Development and Evaluation (GRADE) methodology ((12)) was used to assess available evidence considered by NACI during guidance development. Following critical appraisal of individual studies, summary tables with ratings of the certainty of evidence using the GRADE methodology were prepared. For analyses comparing a two-dose to a three-dose HPV vaccine schedule, a methodology informed by A MeaSurement Tool to Assess systematic Reviews (AMSTAR 2) ((13)) was used to assess available evidence considered by NACI during guidance development. Detailed information regarding the methodology used in the update of this review can be found elsewhere.

## Results

### Efficacy/effectiveness against HPV infection

**Figure 1:**
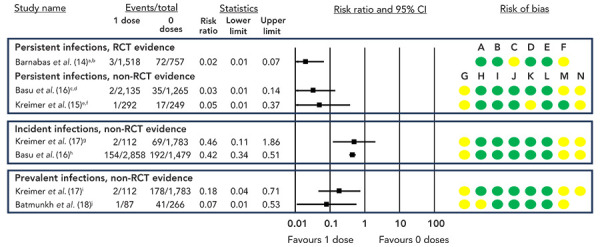
Risk ratios and 95% CI for persistent, prevalent, and incident HPV vaccine-type infections, one dose compared to no doses^a,b,c,d,e,f,g,h,i,j^ Abbreviations: CI, confidence interval; HPV, human papilloma virus; RCT, randomized controlled trial ^a^ Persistent infection defined as detection of a vaccine-type HPV infection at two consecutive visits after the three-month visit, which were obtained no less than four months apart ^b^ Three-year follow-up, nonavalent vaccine effectiveness (VE)=98.8% (95% CI: 91.3%–99.8%), bivalent VE=97.5% (95% CI: 90.0%–99.4%) ^c^ Persistent infection defined as detection of vaccine-type infection in two consecutive samples taken at least 10 months apart ^d^ 10-year follow-up, VE=95.4% (95% CI: 85.0%–99.9%) ^e^ Persistent infection defined as two or more vaccine-type positive tests at least 300 days apart, with no intervening negatives ^f^ Four-year follow-up, VE=95.1% (95% CI: 73.2%–99.8%) ^g^ 11-year follow-up, VE=53.9% (95% CI: -57.1%–92.4%) ^h^ 10-year follow-up, VE=63.5% (95% CI: 52.1%–73.1%) ^i^ 11-year follow-up, VE=82.1% (95% CI: 40.2%–97.0%) ^j^ Six-year follow-up; adjusted prevalence ratio 0.10 (0.01–0.73) Risk of bias legend: A) risk of bias arising from the randomization process, B) risk of bias due to deviations from the intended interventions, C) risk of bias due to missing outcome data, D) risk of bias in measurement of the outcome, E) risk of bias in selection of the reported result, F) overall risk of bias, G) bias due to confounding, H) bias in selection of participants into the study, I) bias in classification of interventions, J) bias due to deviation from intended interventions, K) bias due to missing data, L) bias in measurement of outcomes, M) bias due to selection of reported result, N) overall risk of bias

A GRADE assessment of the available randomized controlled trial (RCT) evidence concluded that a one-dose HPV vaccine schedule resulted in a large reduction in persistent infection compared to no vaccine (high certainty of evidence; **Table 2**). Currently, the KENya Single-dose HPV-vaccine Efficacy (KEN SHE) trial represents the sole RCT evidence demonstrating the efficacy of a single-dose schedule ((14)). This trial randomized women aged 15–20 years (n=2,275) to one dose of either GARDASIL®9, CERVARIX®, or meningococcal vaccine. After three years of follow-up, vaccine effectiveness (VE) against persistent HPV16/18 infection was 97.5% (95% CI: 90.0%–99.4%) and 98.8% (95% CI: 91.3%–99.8%) for GARDASIL®9 and CERVARIX®, respectively. Similar results were seen in the non-RCT evidence, with a single dose probably resulting in reductions in persistent ((15,16)), incident ((16,17)), and prevalent ((17,18)) HPV infections compared to no vaccination (moderate certainty of evidence; Table 2, **Figure 1**).

**Table 2:** Summary of findings comparing one dose to no doses of HPV vaccine

Number of studies	Study design	Number of events/number of participants		Effect		Certainty of evidence	Comments
		Zero doses	One dose	Relative effect (95% CI)	Absolute effect (95% CI)		
**Persistent HPV infection with vaccine types (follow-up ranging from 3–10 years)**							
1 (14)	RCT^a^	72/757 (9.5%)	3/1,518 (0.2%)	RR 0.02 (0.01–0.07)	94 fewer per 1,000 (94 fewer to 88 fewer)	High	A single dose of HPV vaccine results in a large reduction in persistent HPV infections compared to no vaccine
2 (15,16)	Post-hoc RCT analysis	A small number of events in the intervention groups across studies (n=292–2,135); high VE was estimated in each study^b^				Moderate^c^	A single dose of HPV vaccine probably results in a large reduction in persistent HPV infections compared to no vaccine
**Prevalent HPV infection with vaccine types (follow-up ranging from 6–11 years)**							
2 (17,18)	1 post-hoc RCT analysis, 1 observational study	A small number of events in the intervention groups across studies (n=87–221); large reductions in infection prevalence associated with a single dose in each study^d^				Moderate^c^	A single dose of HPV vaccine probably results in reduction in prevalent HPV infections compared to no vaccine
**Incident HPV infection with vaccine types (follow-up ranging from 10–11 years)**							
2 (16,17)	Post-hoc RCT analysis	Number of events dissimilar between studies (n=112–2,858); however, similar reductions in risk compared to unvaccinated were observed^e^				Moderate^c^	A single dose of HPV vaccine probably results in reduction in incident HPV infections compared to no vaccine
**Antibody titres (follow-up ranging from 4–10 years)**							
3 (18–20)	Observational	Varying number of participants in each study (n=30–324), with differing lengths of follow-up and magnitudes of effect across studies; however, the direction of effect was consistent across studies^f^				High	A single dose of HPV vaccine results in an increased immune response compared to no vaccine
**Anogenital warts (follow-up of approximately 2.5 years)**							
1 (21)	Observational	523/52,779 (1.0%)	69/9,898 (0.7%)	aHR^g^ 0.32 (0.20–0.52)	7 fewer per 1,000 (8 fewer to 5 fewer)	Moderate^h^	A single dose of HPV vaccine probably reduces the risk of anogenital warts compared to no vaccine
**Juvenile-onset recurrent respiratory papillomatosis (JoRPP)**							
N/A	N/A	N/A	N/A	N/A	N/A	N/A	N/A

Abbreviations: aHR, adjusted hazard ratio; aVE, adjusted vaccine effectiveness; CI, confidence interval; HPV, human papilloma virus; N/A, not applicable; RCT, randomized controlled trial; RR, relative risk; VE, vaccine effectiveness

^a^ The groups receiving a single dose of GARDASIL®9 and CERVARIX® were collapsed into a single group for the purpose of the analysis

^b^ Consistent results were observed across studies, with both included studies estimating single-dose VE to be similarly high. One study estimated VE at 95.1% (95% CI: 73.2%–99.8%) and the other estimated aVE (adjusted for disease-risk score) at 93.4% (95% CI: 81.1%–99.1%). The definition of persistent infection was similar across studies

^c^ Downgraded by one level due to some concerns with bias due to confounding and selection of the reported result

^d^ Consistent results were observed across studies. A post-hoc analysis of an RCT estimated single-dose VE at 82.1% (95% CI: 40.2%–97.0%). A retrospective observational study did not provide an estimate of VE; however, the adjusted prevalence ratio of HPV infections (adjusted for employment status and income) was 0.08 (95% CI: 0.01%–0.56%) compared to unvaccinated individuals. The definition of prevalent infection was consistent across studies

^e^ Although the risk of incident infection with a single dose was dissimilar between the two included studies (1.8% vs. 5.4%), consistent reductions in risk were observed when compared to unvaccinated individuals. Both included studies estimated similar VE, with one study estimating VE at 53.9% (95% CI: −57.1%–92.4%) and the other estimating aVE (adjusted for disease-risk score) at 54.1% (95% CI: 41.8%–64.1%). The definition of incident infection was consistent across studies

^f^ Magnitude of effect differs across studies; however, this is potentially explained by the differences in study populations and differing lengths of follow-up. In addition, the direction of effect is consistent across studies

^g^ Hazard ratio was adjusted for race/ethnicity, health plan (site), age at enrollment in the health plan, age at beginning of study period, age at first evidence of probable sexual activity (as defined by Healthcare Effectiveness Data and Information Set criteria), age at first dose of HPV vaccine (or proxy date), months enrolled in the health plan, Medicaid enrollment, oral contraceptive use, or history of tests for pregnancy, chlamydia or gonorrhea

^h^ Downgraded by one level due to some concerns with bias, including due to confounding, selection of participants into the study and selection of the reported result

**Figure 2:**
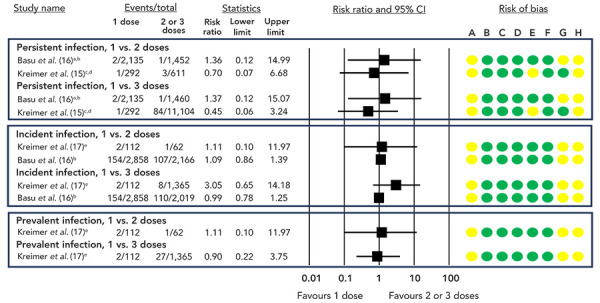
Risk ratios and 95% CI for persistent, prevalent and incident HPV vaccine-type infections, one dose compared to either two or three doses^a,b,c,d,e^ Abbreviations: CI, confidence interval; HPV, human papilloma virus; RCT, randomized controlled trial ^a^ Persistent infection defined as detection of vaccine-type infection in two consecutive samples taken at least 10 months apart ^b^ 10-year follow-up ^c^ Persistent infection defined as two or more vaccine-type positive tests at least 300 days apart, with no intervening negatives ^d^ Four-year follow-up ^e^ 11-year follow-up Risk of bias legend: A) bias due to confounding, B) bias in selection of participants into the study, C) bias in classification of interventions, D) bias due to deviation from intended interventions, E) bias due to missing data, F) bias in measurement of outcomes, G) bias due to selection of reported result, H) overall risk of bias

**Figure 3:**
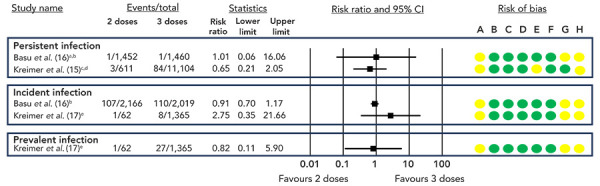
Risk ratios and 95% CI for persistent, prevalent and incident HPV infections, two doses compared to three doses^a,b,c,d,e^ Abbreviations: CI, confidence interval; HPV, human papilloma virus; RCT, randomized controlled trial ^a^ Persistent infection defined as detection of vaccine-type infection in two consecutive samples taken at least 10 months apart ^b^ 10-year follow-up ^c^ Persistent infection defined as two or more vaccine-type positive tests at least 300 days apart, with no intervening negatives ^d^ Four-year follow-up ^e^ 11-year follow-up Risk of bias legend: A) bias due to confounding, B) bias in selection of participants into the study, C) bias in classification of interventions, D) bias due to deviation from intended interventions, E) bias due to missing data, F) bias in measurement of outcomes, G) bias due to selection of reported result, H) overall risk of bias

A GRADE assessment of the available evidence concluded that, compared to two or three doses, there may be little to no difference in persistent, incident, or prevalent HPV infection risk with a one-dose HPV vaccine schedule (low certainty of evidence, **Table 3** and **Table 4**, **Figure 2** and **Figure 3**). Two RCTs evaluating the effectiveness of a two and/or three-dose HPV vaccine schedule have conducted post-hoc analyses to also estimate the VE of a one-dose schedule, with both studies reporting similar VE across all dosing schedules, up to 10 ((16)) or 11 years ((17)) (low certainty of evidence; Table 3 and Table 4, Figure 2 and Figure 3).

**Table 3:** Summary of findings comparing one dose to two doses of HPV vaccine

Number of studies	Study design	Number of events/number of participants		Effect		Certainty of evidence	Comments
		Two doses	One dose	Relative effect (95% CI)	Absolute effect (95% CI)		
**Persistent HPV infection with vaccine types (follow-up ranging from 4–10 years)**							
2 (15,16)	Post-hoc RCT analysis	A small number of events in both the intervention (n=292–2,135) and control arms (n=611–1,452) across studies; high VE estimated for both arms in each study^a^				Low^b,c^	A single dose of HPV vaccine may result in little to no difference in persistent HPV infections compared to two doses
**Prevalent HPV infection with vaccine types (follow-up of 11 years)**							
1 (17)	Post-hoc RCT analysis	1/62 (1.6%)	2/112 (1.8%)	RR 1.11 (0.10–11.97)	2 more per 1,000 (15 fewer to 177 more)	Low^d,e^	A single dose of HPV vaccine may result in little to no difference in prevalent HPV infections compared to two doses
**Incident HPV infection with vaccine types (follow-up ranging from 10–11 years)**							
2 (16,17)	Post-hoc RCT analysis	Number of events dissimilar between studies (n [one dose]=112–2,858; n [two doses]=62–2,166), as the baseline risk of events varies across studies; however, VE estimates for each group are similar across studies^f^				Low^d,e^	A single dose of HPV vaccine may result in little to no difference in incident HPV infections compared to two doses
**Antibody titres (follow-up ranging from 2–16 years)**							
1 (22)	RCT	310	310	Ratio of GMTs ranging from 0.11 (0.09–0.14) to 0.21 (0.16–0.26)	N/A	High	A single dose of HPV vaccine results in a decreased immune response compared to two doses
2 (21,23)	Post-hoc RCT analysis	Dissimilar number of participants between intervention and control arms, across all studies; however, consistent magnitude and direction of effect across studies				High	A single dose of HPV vaccine results in a decreased immune response compared to two doses
**Histological and cytological abnormalities (follow-up of 10 years)**							
1 (16)	Post-hoc RCT analysis	1/1,128 (0.9%)	4/1,511 (2.6%)	RR 2.99 (0.33–26.80)	2 more per 1,000 (1 fewer to 23 more)	Low^b,e^	A single dose of HPV vaccine may result in little to no difference in cervical abnormalities compared to two doses
**CIN2+ (follow-up of 10 years)**							
1 (16)	Post-hoc RCT analysis	0/1,128 (0%)	0/1,511 (0%)	Not estimable	Not estimable	Low^b,g^	A single dose of HPV vaccine may result in little to no difference in CIN2+ compared to two doses
**HPV-associated cancer (follow-up of 10 years)**							
1 (16)	Post-hoc RCT analysis	0/1,128 (0%)	0/1,511 (0%)	Not estimable	Not estimable	Very low^b,h,i^	Data insufficient to determine association
**Anogenital warts (follow-up of approximately 2.5 years)**							
1 (21)	Observational	42/8,046 (0.5%)	69/9,898 (0.7%)	aHR^j^ 0.74 (0.35–1.60)	2 more per 1,000 (5 fewer to 4 more)	Low^e,k^	A single dose of HPV vaccine may result in little to no difference in the risk of anogenital warts compared to two doses
**Juvenile-onset recurrent respiratory papillomatosis (JoRPP)**							
N/A	N/A	N/A	N/A	N/A	N/A	N/A	N/A

Abbreviations: aHR, adjusted hazard ratio; CI, confidence interval; CIN, cervical intraepithelial neoplasia; GMT, geometric mean titre; HPV, human papilloma virus; N/A, not applicable; RCT, randomized controlled trial; RR, relative risk; VE, vaccine effectiveness

^a^ Consistent results observed across studies, with both included studies estimating one and two-dose VE to be similarly high. One study estimated VE at 93.4% (95% CI: 81.1%–99.1%) and 93.7% (95% CI: 78.9%–99.8%) and the other estimated VE at 95.1% (95% CI: 73.2%–99.8%) and 89.6% (95% CI: 68.9%–97.5%) for one and two-dose groups, respectively. The definition of persistent infection was similar across studies

^b^ Downgraded one level due to some concerns with bias due to confounding and selection of the reported result

^c^ Downgraded one level due to imprecision, few events and a 95% CI that encompasses a potential benefit, no effect, and a potential harm

^d^ Downgraded one level due to some concerns with bias due to confounding

^e^ Downgraded one level due to imprecision and a 95% CI that encompasses a potential benefit, no effect, and a potential harm

^f^ The baseline risk of incident infection for both one and two doses was dissimilar across the two included studies. However, VE estimated for both the one and two-dose arms of the trials was similar across studies, with one study reporting VE of 54.1% (95% CI: 41.8%–64.1%) and 59% (95% CI: 46.9%–69.1%) and the other reporting VE of 53.9% (95% CI: −57.1%–92.4%) and 58.4% (95% CI: −110.9%–97.9%) for one and two-dose groups, respectively

^g^ Downgraded by one level due to imprecision, as the optimal information size was not met

^h^ Downgraded by two levels due serious concerns over indirectness, due to a small number of events, owing to the follow-up period. A 10-year follow-up is insufficient to determine the effect on cancer incidence

^i^ Downgraded by one level due to some concerns over imprecision. There are no events and the optimal information size was not met

^j^ Hazard ratio was adjusted for race/ethnicity, health plan (site), age at enrollment in the health plan, age at beginning of study period, age at first evidence of probable sexual activity (as defined by Healthcare Effectiveness Data and Information Set criteria), age at first dose of HPV vaccine (or proxy date), months enrolled in the health plan, Medicaid enrollment, oral contraceptive use, or history of tests for pregnancy, chlamydia, or gonorrhea

^k^ Downgraded by one level due to some concerns with bias, including due to confounding, selection of participants into the study and selection of the reported result

**Table 4:** Summary of findings comparing one dose to three doses of HPV vaccine

Number of studies	Study design	Number of events/number of participants		Effect		Certainty of evidence	Comments
		Three doses	One dose	Relative effect (95% CI)	Absolute effect (95% CI)		
**Persistent HPV infection with vaccine types (follow-up ranging from 4–10 years)**							
2 (15,16)	Post-hoc RCT analysis	A small number of events in both the intervention (n=292–2,135) and control (n=1,460–11,104) arms across studies; high VE estimated for both arms in each study^a^				Low^b,c^	A single dose of HPV vaccine may result in little to no difference in persistent HPV infections compared to three doses
**Prevalent HPV infection with vaccine types (follow-up of 11 years)**							
1 (17)	Post-hoc RCT analysis	27/1,365 (2.0%)	2/112 (1.8%)	RR 0.90 (0.22–3.75)	2 fewer per 1,000 (15 fewer to 54 more)	Low^d,e^	A single dose of HPV vaccine may result in little to no difference in prevalent HPV infections compared to three doses
**Incident HPV infection with vaccine types (follow-up ranging from 10–11 years)**							
2 (16,17)	Post-hoc RCT analysis	Number of events dissimilar between studies (n [one dose]=112–2,858; n [two doses]=1,365–2,019), as the baseline risk of events varies across studies^f^				Low^d,e^	A single dose of HPV vaccine may result in little to no difference in incident HPV infections compared to three doses
**Antibody titres (follow-up ranging from 2–16 years)**							
1 (22)	RCT	310	310	Ratio of GMTs ranging from 0.06 (0.05–0.07) to 0.19 (0.15–0.24)	N/A	High	A single dose of HPV vaccine results in a decreased immune response compared to three doses
2 (21,23)	Post-hoc RCT analyses	Dissimilar number of participants between intervention and control arms, across all studies; however, consistent magnitude and direction of effect across studies				High	A single dose of HPV vaccine results in a decreased immune response compared to three doses
**Histological and cytological abnormalities (follow-up of 10 years)**							
1 (16)	Post-hoc RCT analysis	1/1,037 (0.9%)	4/1,511 (2.6%)	RR 2.75 (0.31–24.53)	2 more per 1,000 (1 fewer to 23 more)	Low^b,e^	A single dose of HPV vaccine may result in little to no difference in cervical abnormalities compared to three doses
**CIN2+ (follow-up of 10 years)**							
1 (16)	Post-hoc RCT analysis	0/1,037 (0%)	0/1,511 (0%)	Not estimable	Not estimable	Low^b,g^	A single dose of HPV vaccine may result in little to no difference in CIN2+ compared to three doses
**HPV-associated cancer (follow-up of 10 years)**							
1 (16)	Post-hoc RCT analysis	0/1,037 (0%)	0/1,511 (0%)	Not estimable	Not estimable	Very low^b,h,i^	Data insufficient to determine association
**Anogenital warts (follow-up of approximately 2.5 years)**							
1 (21)	Observational	91/57,287 (0.2%)	69/9,898 (0.7%)	aHR^j^ 0.63 (0.37–1.09)	3 more per 1,000 (1 fewer to 4 more)	Low^e,k^	A single dose of HPV vaccine may result in little to no difference in the risk of anogenital warts compared to three doses
**Juvenile-onset recurrent respiratory papillomatosis (JoRPP)**							
N/A	N/A	N/A	N/A	N/A	N/A	N/A	N/A

Abbreviations: aHR, adjusted hazard ratio; CI, confidence interval; CIN, cervical intraepithelial neoplasia; GMT, geometric mean titre; HPV, human papilloma virus; N/A, not applicable; RCT, randomized controlled trial; RR, relative risk; VE, vaccine effectiveness

^a^ Consistent results observed across studies, with both included studies estimating one and three-dose VE to be similarly high. One study estimated VE at 93.4% (95% CI: 81.1%–99.1%) and 90.3% (95% CI: 71.9%–98.5%) and the other estimated VE at 95.1% (95% CI: 73.2%–99.8%) and 87.0% (95% CI: 83.7%–89.7%) for one- and three-dose groups, respectively. The definition of persistent infection was similar across studies

^b^ Downgraded one level due to some concerns with bias due to confounding and selection of the reported result

^c^ Downgraded one level due to imprecision, few events and a 95% CI that encompasses a potential benefit, no effect, and a potential harm

^d^ Downgraded one level due to some concerns with bias due to confounding

^e^ Downgraded one level due to imprecision and a 95% CI that encompasses a potential benefit, no effect, and a potential harm

^f^ The baseline risk of incident infection for both one and three doses was dissimilar across the two included studies, as was the estimated VE for one of the included studies. The VE estimated for the one and two-dose arms of the trials was 54.1% (95% CI: 41.8%–64.1%) and 54.7% (95% CI: 40.9%–65.0%) in one study and 53.9% (95% CI: −57.1%–92.4%) and 84.9% (95% CI: 69.8%–93.2%) in the other, for one and two-dose groups, respectively. The difference in VE reported can be at least partially explained by the small number of events and participants in the one-dose group

^g^ Downgraded by one level due to imprecision, as the optimal information size was not met

^h^ Downgraded by two levels due serious concerns over indirectness, due to a small number of events, owing to the follow-up period. A 10-year follow-up is insufficient to determine the effect on cancer incidence

^i^ Downgraded by one level due to some concerns over imprecision. There are no events, and the optimal information size was not met

^j^ Hazard ratio was adjusted for race/ethnicity, health plan (site), age at enrollment in the health plan, age at beginning of study period, age at first evidence of probable sexual activity (as defined by Healthcare Effectiveness Data and Information Set criteria), age at first dose of HPV vaccine (or proxy date), months enrolled in the health plan, Medicaid enrollment, oral contraceptive use, or history of tests for pregnancy, chlamydia or gonorrhea

^k^ Downgraded by one level due to some concerns with bias, including due to confounding, selection of participants into the study and selection of the reported result

The Costa Rica Vaccine Trial (CVT) was originally designed to test the efficacy of a three-dose schedule of CERVARIX® in females aged 18–25 years (compared to control hepatitis A vaccine); however, approximately 20% of participants did not complete their three-dose schedule, primarily due to pregnancy or colposcopy referral, thus creating cohorts who received a one or two-dose schedule. After 11 years of follow-up, VE against prevalent HPV16/18 infection was similar among recipients of either one-dose (82.1%; 95% CI: 40.2%–97.0%), two-dose (83.8%; 95% CI: 19.5%–99.2%) or three-dose (80.2%; 95% CI: 70.7%–87.0%) schedules ((17)).

Similar results were also seen in the International Agency for Research on Cancer (IARC) study from India, which was originally designed to compare two and three doses of GARDASIL® in females aged 10–18 years. However, numerous participants did not complete their full vaccine schedule, as recruitment of girls into HPV trials was suspended by the Indian government in 2010. Vaccine effectiveness against persistent HPV16/18 infection was similar among women who received one (95.4%; 95% CI: 85%–99.1%), two (93.1%; 95% CI: 77.3%–99.8%) or three (93.3%; 95% CI: 77.5%–99.7%) doses after 10 years of follow-up ((16)).

### Efficacy/effectiveness against cervical precancerous lesions

Among included studies, only the IARC trial (16) reported data on the effect of different HPV vaccine schedules on cervical precancers and HPV-related cancers. After 10 years of follow-up, 16/4,626 (0.3%) of unvaccinated women reported cervical intraepithelial neoplasia (CIN) grade 1, compared to 4/1,511 (0.3%), 1/1,128 (0.1%) and 1/1,037 (0.1%) in the one, two and three-dose groups, respectively. There were no cases of CIN2 or greater in any of the vaccine groups, regardless of the number of doses received, while 5/4,626 women (0.1%) in the unvaccinated group experienced CIN2 or greater. Additionally, there were no cases of HPV-related cancers in any of the groups.

A GRADE assessment of the available evidence concluded that there may be little to no difference in the risks of cervical abnormalities or CIN2+ between one and either two or three-dose schedules (low certainty of evidence; Table 3 and Table 4).

### Efficacy/effectiveness against anogenital warts

There is currently no clinical trial evidence comparing the effect of a single dose to either two or three doses on the risk of AGW. However, an observational study from the United States (n=64,517) compared the risk of AGW in female participants who received one dose to those who received no, two, or three doses of GARDASIL® ((21)). Propensity score-weighted incidence rates were 761.9 (95% CI: 685.5–849.1), 256.6 (95% CI: 161.8–432.3), 194.2 (95% CI: 108.0–386.4), and 161.8 (95% CI: 124.4–214.6) per 100,000 person-years in the unvaccinated, one, two and three-dose groups, respectively. Propensity score-weighted hazard ratios (HRs) demonstrated no statistically significant difference between the groups, with HRs of 0.74 (95% CI: 0.35–1.60) and 0.63 (95% CI: 0.37–1.09) for two and three doses (compared to one), respectively (no direct comparison of the two and three-dose groups).

A GRADE assessment of the available evidence concluded that a single dose of HPV vaccine probably reduces the risk of AGW compared to no vaccine (moderate certainty of evidence; Table 2), and that there may be little to no difference in risk, compared to a two or three-dose schedule (low certainty of evidence; Table 3 and Table 4).

### Antibody titres

**Figure 4:**
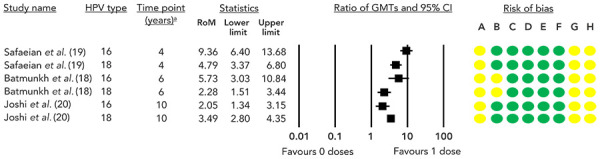
Ratio of geometric mean titres and 95% CI comparing one dose to zero doses^a^ Abbreviations: CI, confidence interval; GMT, geometric mean titre; HPV, human papilloma virus; RCT, randomized controlled trial; RoM, ratio of means ^a^ Only the final timepoint provided by each study is displayed Risk of bias legend: A) bias due to confounding, B) bias in selection of participants into the study, C) bias in classification of interventions, D) bias due to deviation from intended interventions, E) bias due to missing data, F) bias in measurement of outcomes, G) bias due to selection of reported result, H) overall risk of bias

**Figure 5:**
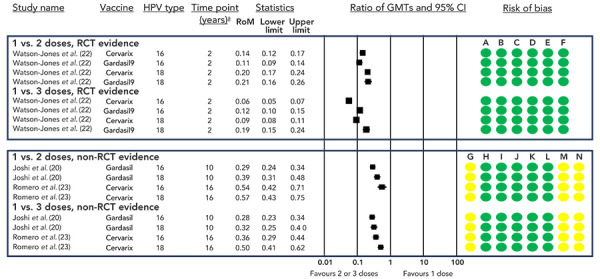
Ratio of geometric mean titres and 95% CI comparing one dose to either two or three doses^a^ Abbreviations: CI, confidence interval; GMT, geometric mean titre; HPV, human papilloma virus; RCT, randomized controlled trial; RoM, ratio of means ^a^ Only the final timepoint provided by each study is displayed Risk of bias legend: A) risk of bias arising from the randomization process, B) risk of bias due to deviations from the intended interventions, C) risk of bias due to missing outcome data, D) risk of bias in measurement of the outcome, E) risk of bias in selection of the reported result, F) overall risk of bias, G) bias due to confounding, H) bias in selection of participants into the study, I) bias in classification of interventions, J) bias due to deviation from intended interventions, K) bias due to missing data, L) bias in measurement of outcomes, M) bias due to selection of reported result, N) overall risk of bias

A GRADE assessment of the available evidence concluded that a single dose of HPV vaccine results in an increased immune response compared to no vaccine ((18–20)) (high certainty of evidence; Table 2, **Figure 4**), and a decreased immune response compared to two or three doses (high certainty of evidence; Table 3 and Table 4, **Figure 5**).

**Figure 6:**
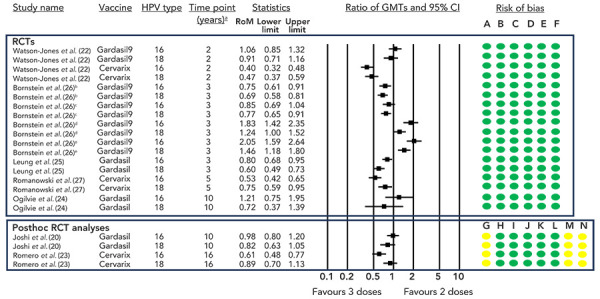
Ratio of geometric mean titres and 95% CI comparing two doses to three doses^a,b,c,d,e^ Abbreviations: CI, confidence interval; GMT, geometric mean titre; HPV, human papilloma virus; RCT, randomized controlled trial; RoM ratio of means ^a^ Only the final timepoint provided by each study is displayed ^b^ Boys, six-month interval ^c^ Girls, six-month interval ^d^ Boys, 12-month interval ^e^ Girls, 12-month interval Risk of bias legend: A) risk of bias arising from the randomization process, B) risk of bias due to deviations from the intended interventions, C) risk of bias due to missing outcome data, D) risk of bias in measurement of the outcome, E) risk of bias in selection of the reported result, F) overall risk of bias, G) bias due to confounding, H) bias in selection of participants into the study, I) bias in classification of interventions, J) bias due to deviation from intended interventions, K) bias due to missing data, L) bias in measurement of outcomes, M) bias due to selection of reported result, N) overall risk of bias

The Dose Reduction Immunobridging and Safety study (DoRIS) from Tanzania randomized females aged 9–14 years (n=930) to receive one, two, or three doses of CERVARIX® or GARDASIL®9 ((22)). Antibody titres were statistically significantly lower for one-dose recipients compared to two or three-dose recipients for both vaccines (Figure 5). However, while lower titres were observed for the one-dose schedule, the antibody response was sustained through year two (end of study). In individuals who received two doses of GARDASIL®9, antibody titres were non-inferior compared to those who received three doses; however, they were significantly lower (and non-inferiority was not met) for those receiving two doses of CERVARIX® (**Figure 6**).

Two post-hoc analyses of the CVT and IARC trials (data up to 16 ((23)) and 10 ((21)), respectively) have produced similar results to the DoRIS study, with a single-dose schedule producing inferior but sustained antibody titres (high certainty of evidence; Table 3 and Table 4, Figure 5).

Several RCTs provide data comparing the antibody titres of a two-dose versus three-dose schedule (Figure 6). The long-term follow-up of a Canadian RCT in girls aged 9–13 years receiving GARDASIL® demonstrated a non-inferior antibody response with two doses for HPV6, HPV11 and HPV16, ten years following vaccination (non-inferiority not met for HPV18) ((24)). Another RCT of girls aged 9–14 years receiving GARDASIL® demonstrated a non-inferior immune response for HPV16 and HPV18, three years following vaccination ((25)). In a multinational RCT using GARDASIL®9, girls aged 9–14 years were randomized to receive two (six or 12 months apart) or three doses (six months apart), while boys aged 9–14 years were randomized to receive two doses six or 12 months apart. While the individuals receiving two doses six months apart had generally lower/similar antibody levels compared to those receiving three doses, those receiving two doses 12 months apart generally had higher/similar antibody levels compared to those given three doses within six months, three years after vaccination ((26)), suggesting the interval between doses may be more important than the number of doses. Lastly, in an RCT of females aged 9–25 years receiving CERVARIX®, HPV16 and HPV18 antibody titres appeared slightly higher after a three-dose schedule compared to a two-dose schedule, regardless of age strata (9–14 and 15–25 years), five years following vaccination. However, no test of non-inferiority was performed ((27)).

## Discussion

The effectiveness/efficacy and immunogenicity of various HPV vaccine schedules were reviewed. Available evidence suggests that single-dose VE against HPV infection may be similar to that of two or three doses. Antibody titres, however, indicate a lower immune response with a single dose compared to two or three doses. Currently, there is no established correlate of protection for HPV, and therefore the clinical relevance of this decreased immune response is unknown. The interpretation of results from other clinical outcomes, such as the risks of CIN and abnormal cytology, remain challenging due to limitations of the included studies. In addition to the currently available evidence outlined above, which includes follow-up for up to 16 years post-immunization depending on the study and clinical protection outcome, longer follow-up data are expected in the coming years from multiple key studies. As trials continue to accrue data, follow-up will remain important as trial participants reach the age of increased baseline risk of cervical abnormalities and associated cancers, as data for these outcomes is currently limited. Two additional RCTs from Costa Rica are underway and are expected to produce estimates of single dose VE in females 12–16 years and 18–30 years of age by 2025 and 2026, respectively ((28,29)).

## Limitations

There are several limitations to the current data. Data are predominately limited to female adolescents and young women, with a primary focus on cervical HPV infection and cervical cancer precursors. However, several additional cancers are attributable to HPV infections (i.e., other anogenital, and head/neck cancers) (2), for which there are currently no data. While there is no clinical trial data on VE of a single vaccine dose in males, several retrospective observational studies include both biological sexes. However, only two studies report results stratified by sex, with neither study reporting a difference in HPV infection risk between dosing schedules in males ((30,31)). Neither study was eligible for inclusion, however, as both were considered at serious risk of bias. It is possible that different antibody levels or immunologic factors are required for protection in the female versus male genital tract, and for protection against warts and head/neck/anal cancer. Future research on the effect of single dose HPV vaccination and other HPV-related cancers, including trials where clinical outcomes are assessed among male populations, will be important for public health decision-making. Data are also currently lacking on the effect of a one-dose schedule in immunocompromised individuals. Only one observational study that provided data for this group was identified, with no difference in the incidence of abnormal cervical cytology observed between dosing schedules in HIV-positive females. This study was, however, considered at serious risk of bias and therefore not eligible for inclusion ((32)).

## Conclusion

Current clinical data on reduced HPV dosing schedules are promising. Longer-term follow-up of clinical trial participants, as well as monitoring real-world outcomes in countries where the change to single-dose schedules have already taken place, can help better understand the duration of protection against HPV infection conferred from reduced dosing schedules. In addition, when considering population-level programmatic changes, several additional factors will likely require consideration, including impacts of a program change on acceptability and uptake of the HPV vaccine, as well as on health inequities and access to the vaccine.
